# Lepidoptera family-group names proposed by Thaddeus William Harris in 1841

**DOI:** 10.3897/zookeys.264.4442

**Published:** 2013-02-06

**Authors:** B. Christian Schmidt, J. Donald Lafontaine

**Affiliations:** 1 Canadian Food Inspection Agency, Canadian National Collection of Insects, Arachnids and Nematodes, K.W. Neatby Bldg., 960 Carling Ave., Ottawa, ON, Canada K1A 0C6; 2 Canadian National Collection of Insects, Arachnids, and Nematodes, Biodiversity Program, Agriculture and Agri-Food Canada, K.W. Neatby Bldg., C.E.F., Ottawa, Ontario, Canada K1A 0C6

**Keywords:** Acronictinae, Agrotini, Bistonini, Boarmiini, Ennominae, Geometridae, Hadenini, Hyberniini, Noctuidae

## Abstract

In 1841, T. W. Harris published “A Report on the Insects of Massachusetts, Injurious to Vegetation,” a seminal work in North American Entomology and one of the first New World publications to describe Lepidoptera species. Although appearing in several subsequent editions, the eight family-group names proposed by Harris were largely overlooked. In summarizing Harris’ family-group names, we show that authorship for two Noctuidae names in current usage require changes: Acronictinae Harris, 1841 (originally as Acronyctadae) has priority over Heinemann, 1859, and Agrotini Harris, 1841 (as Agrotitidae) has priority over Rambur, 1848. Mamestradae Harris, 1841 is also a senior synonym of Mamestrinae Hampson, 1902 (Type genus: *Mamestra* Ochsenheimer, 1816), an available name that is currently a junior subjective synonym of Noctuidae: Noctuinae: Hadenini Guenée, 1837 (Type genus: *Hadena* Schrank, 1802). Geometridae: Ennominae: Hyberniini Harris, 1841 (as Hyberniadae), based on *Erranis* Hübner, [1825] (= *Hybernia* Berthold, 1827), has precedence over two family-group names with long-standing usage, Bistonini Stephens, 1850 and Boarmiini Duponchel, 1845, and a reversal of precedence for the latter two names over Hyberniini is proposed under ICZN guidelines.

## Introduction

In one of the earliest compendia of North American entomology, Thaddeus William Harris’ “A Report on the Insects of Massachusetts, Injurious to Vegetation” describes the life histories of hundreds of insect species, often in extensive detail. Over a dozen Lepidoptera species were described and named by Harris therein, including familiar species such as *Lophocampa maculata*, *Acronicta americana*, and *Euxoa messoria*. First published in 1841, the “Report” was re-printed in 1842 (Harris 1842), and subsequently released in a 2^nd^ (Harris 1852) and 3^rd^ edition (Harris 1862). The 3^rd^ edition was published after Harris’ death in 1856, and included the addition of butterfly species based on Harris’ manuscripts, including descriptions of 10 new species. Harris described over 50 new taxa, 40 of which are currently valid species names.

Less well-known are the family-group names proposed by Harris in 1841, only two of which are currently attributed to him – Lasiocampidae (Lasiocampoidea) and Ceratocampinae (Bombycoidea: Saturniidae). In an earlier work on sphinx moths (Harris 1839), Harris named the Macroglossiadae, currently Sphingidae: Macroglossinae. The purpose of this paper is to review the eight family-group names proposed by Harris (1841). Authorship for two family-group names in current usage should be attributed to Harris in accordance with publication priority, viz. Acronictinae Harris, 1841 (*nec*. Heinemann, 1859) and Agrotini Harris, 1841 (*nec*. Rambur, 1848). The remaining Harris names are junior homonyms or junior subjective synonyms; one is proposed as a *nomen oblitum*.

## Family-group names and ICZN

Harris classified the Lepidoptera similar to the categories proposed by Linnaeus, where the non-sphingid moths were further divided into seven groups, the Bombyces, Noctuae, Geometrae, Pyralides, Tortrices, Tineae and Alucitae. Harris (1841) proposed new family-group names in the Bombyces (Liparidae, Lasiocampadae, Ceratocampadae), Noctuae (Notodontadae, Acronyctadae, Agrotitidae, Mamestradae), and Geometrae (Hyberniadae). He indicated new family-group names by stating each “may be called” or “which I call,” and indicated that the name was based on what he considered the corresponding “core genus.” This prose was not followed for family names he knew were already named, such as the Lithosiadae (p. 240), and the Nonagriadae(p. 318), both used by Harris in 1833 along with other previously established family-group names. Eight family-group names were proposed by Harris in 1841 (and not included by him in his 1833 work), and we compared these to those summarized by Speidel and Naumann (2005) and Fibiger and Lafontaine (2005) for the Noctuoidea, and Forum Herbulot (2003) for the Geometroidea.

## Family-group names proposed by Harris, 1841

Original spelling of family-group names is given in bold, followed by the current taxonomic position and emended spelling in square brackets.

**Liparidae** (p. 260) [Lymantriidae Hampson, 1893]. A junior synonym of Liparides Boisduval, 1834. Both family-group names are invalid due to homonymy of the type genus, *Liparis* Ochsenheimer, 1810 (= *Liparis* Scopoli, 1777 [Pisces]).

**Lasiocampadae** (p. 265) [Lasiocampoidea: Lasiocampidae Harris, 1841]. Correctly attributed to Harris in recent publications, e.g., Franclemont (1973: 25), de Freina and Witt (1987: 33).

**Ceratocampadae** (p. 287) [Saturniidae: Ceratocampinae Harris, 1841]. Correctly attributed to Harris in recent works (e.g., Tuskes et al. 1996), but Ferguson (1971: 18) considered Citheroniinae Neumoegen and Dyar, 1894 to be the valid, although junior, name.

**Acronyctadae** (p. 316) [Noctuidae: Acronictinae Harris, 1841]. A senior synonym of Acronyctadae Heinemann, 1859.

**Agrotitidae** (p. 321) [Noctuidae: Noctuinae: Agrotini Harris, 1841]. A senior synonym of Agrotides Rambur, 1848, currently ranked as a tribe (e.g., Lafontaine 2004, Fibiger and Lafontaine 2005).

**Mamestradae** (p. 329) [Noctuidae: Noctuinae: Hadenini Guenée, 1837]. A senior synonym of Mamestrinae Hampson, 1902. Mamestrini (type genus: *Mamestra* Ochsenheimer, 1816) is currently considered a junior subjective synonym of Hadenini Guenée, 1837 (as Hadenidi), e.g., Fibiger and Lafontaine (2005).

**Herminiadae** (p. 344) [Erebidae: Herminiinae Leach, [1815]]. A junior synonym of Herminida Leach, [1815].

**Hyberniadae** (p. 332) [Geometridae: Ennominae: Boarmiini Duponchel, 1845]. *Hybernia* Berthold, 1827 is a junior objective synonym of *Erannis* Hübner, [1825] (Ferguson 1983). Hyberniini Harris has priority over Hyberniini Duponchel, 1845 (as Hibernites), and also over Boarmiini Duponchel, 1845 (as Boarmites) and Bistonini Stephens, 1850 (as Bistonidi). Although mostly treated as a valid tribe prior to Holloway (1993), Bistonini has recently been subsumed within Boarmiini (e.g., Holloway 1993, Sihvonen et al. 2011). Hyberniini was used (as Hybernites) by Bruand (1846), Stephens (1850) and Guenée (1857), but has apparently not been used as a family-group name after 1899 and therefore meets requirement 23.9.1.1 for “Reversal of Precedence” under ICZN rules. Both Boarmiini and Bistonini have long-standing usage in the literature, summarized in Table 1, and thereby meet the second requirement (23.9.1.2) for “Reversal of Precedence” (ICZN), which states that the name in question should be cited in at least 25 works, published by at least 10 authors in the immediately preceding 50 years and encompassing a span of not less than 10 years. The Geometridae family-group names (Forum Herbulot 2003) in question should therefore be revised as follows:

Boarmiini Duponchel, 1845(Boarmites) *nomen protectum*

= Hyberniini Harris, 1841 (Hyberniadae) *nomen oblitum*

= Hyberniini Duponchel, 1845 (Hibernites) junior synonym of Hyberniadae Harris, 1841

= Bistonini Stephens, 1850 (Bistonidi)

**Table 1.** Publications citing Boarmiini or Bistonini as a family-group name.

**Boarmiini**	**Bistonini**
Brehm and Fiedler 2003, 2004, 2005; Ferguson 1983, 2008; Forbes 1948; Holloway 1993, 2011; Krüger 2007; McGuffin 1977, 1981, 1987; Minet and Scoble 1998; Õunap et al. 2011; Patočka 1986, 1993; Pitkin 2002; Pohl et al. 2010; Rindge 1972, 1976; Riotte 1992; Sihvonen et al. 2011; Stephens and Gibbs 2003; Vargas 2007, 2010; Viidalepp et al. 2007; Wahlberg et al. 2010; Young 2003.	Butler 1986; Ferguson 1983; Ferris 2010; Forbes 1948; Hackray et al. 1984; Holloway 1993; Hunter 1995; McGuffin 1977, 1981, 1987; Miller 1996; Minet and Scoble 1998; Õunap et al. 2011; Patočka 1978; Pellmyr 1980; Pitkin 2002; Pohl et al. 2010; Powell and Opler 2009; Rindge 1975, 1985; Riotte 1992; Rose 1985; Viidalepp 1989; Viidalepp et al. 2007; Wahlberg et al. 2010.
